# P-842. Community-onset vs. Hospital-onset *Staphylococcus aureus* Bacteremia: Implications for Patients and Healthcare Systems

**DOI:** 10.1093/ofid/ofae631.1034

**Published:** 2025-01-29

**Authors:** Juan Carlos Rico Crescencio, Ryan K Dare, Jordan Myers

**Affiliations:** University of Arkansas for Medical Sciences, College of Medicine, Little Rock, Arkansas; University of Arkansas for Medical Sciences, College of Medicine, Little Rock, Arkansas; University of Arkansas for Medical Sciences, Little Rock, Arkansas

## Abstract

**Background:**

*Staphylococcus aureus* is a leading cause of bacteremia, associated with high rates of morbidity and mortality. It poses a tremendous burden to the healthcare system in terms of cost and resource utilization. This study's objective is to evaluate the incidence and clinical outcomes of both community and hospital-onset *S. aureus* bacteremia (SAB).

**Methods:**

This study is a retrospective analysis of adult patients with SAB at an academic medical center in Arkansas from May 2016 to January 2024. Subjects were identified as hospital-onset SAB (HO-SAB) if positive blood cultures were collected ≥ 48 hours after admission or community-onset SAB (CO-SAB) if blood cultures were collected < 48 hours after admission.

**Results:**

A total of 1,179 patients had SAB during the study period of which 230 (19.5%) died or transitioned to hospice care. In-house mortality varied by age(11%< 35y vs 48%≥ 85 y). There were similar rates of mortality between patients with MRSA (21%) and MSSA (19%) bacteremia. Infectious disease (ID) consultation was common (N=1,013; 86%) and resulted in a lower mortality rate compared to patients without ID consultation (16.2% vs 39.8%; P< 0.001). There were 254 (21%) HO-SAB cases with an incidence of 0.22 episodes per 1,000 patient days. HO-SAB incidence increased following the onset of the COVID-19 pandemic (0.17 vs 0.28 cases per 1,000 patient days). In addition, in-house mortality of patients with HO-SAB also increased following the onset of the COVID-19 pandemic (20.8% vs 32.8%). Patients with HO-SAB had a longer median length of stay (20.8 days vs 8.9 days; P< 0.001) and a higher rate of in-hospital mortality (27.2% vs 17.4%; P< 0.001) compared to patients with CO-SAB.

**Conclusion:**

This study highlights the tremendous impact of SAB for patients and the healthcare system. SAB mortality remained high however, ID consultation resulted in significantly lower mortality rates. HO-SAB is unfortunately common and patients with HO-SAB had longer LOS and a higher rate of in-house mortality compared to patients with CO-SAB. Following the onset of the pandemic, HO-SAB incidence and in-hospital mortality increased. Further studies are necessary to understand the risk factors and prevention of HO-SAB.

**Disclosures:**

**All Authors**: No reported disclosures

